# P-1353. Earlier Cefiderocol Use was Associated with Better Outcomes for Challenging Gram-Negative Bacterial Infections: US Results From the Global Observational PROVE Study

**DOI:** 10.1093/ofid/ofaf695.1541

**Published:** 2026-01-11

**Authors:** Cornelius J Clancy, Anne Lachiewicz, Stefano Verardi, Karan Gill, Anne Santerre Henriksen, Sean T Nguyen

**Affiliations:** University of Pittsburgh, Pittsburgh, Pennsylvania; University of North Carolina, Chapel Hill, NC; Shionogi, B.V., London, England, United Kingdom; Shionogi, London, England, United Kingdom; Shionogi BV, London, UK, London, England, United Kingdom; Shionogi Inc., Florham Park, NJ

## Abstract

**Background:**

The PROVE study enrolled 508 patients in the USA with serious Gram-negative bacterial infections treated with cefiderocol in routine clinical practice. The clinical outcomes of this subset of patients were assessed.

**Figure:**
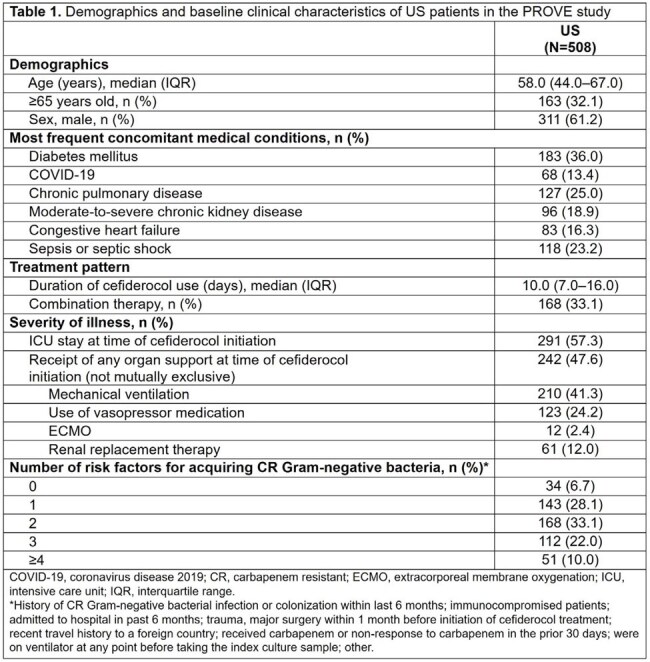

**Figure:**
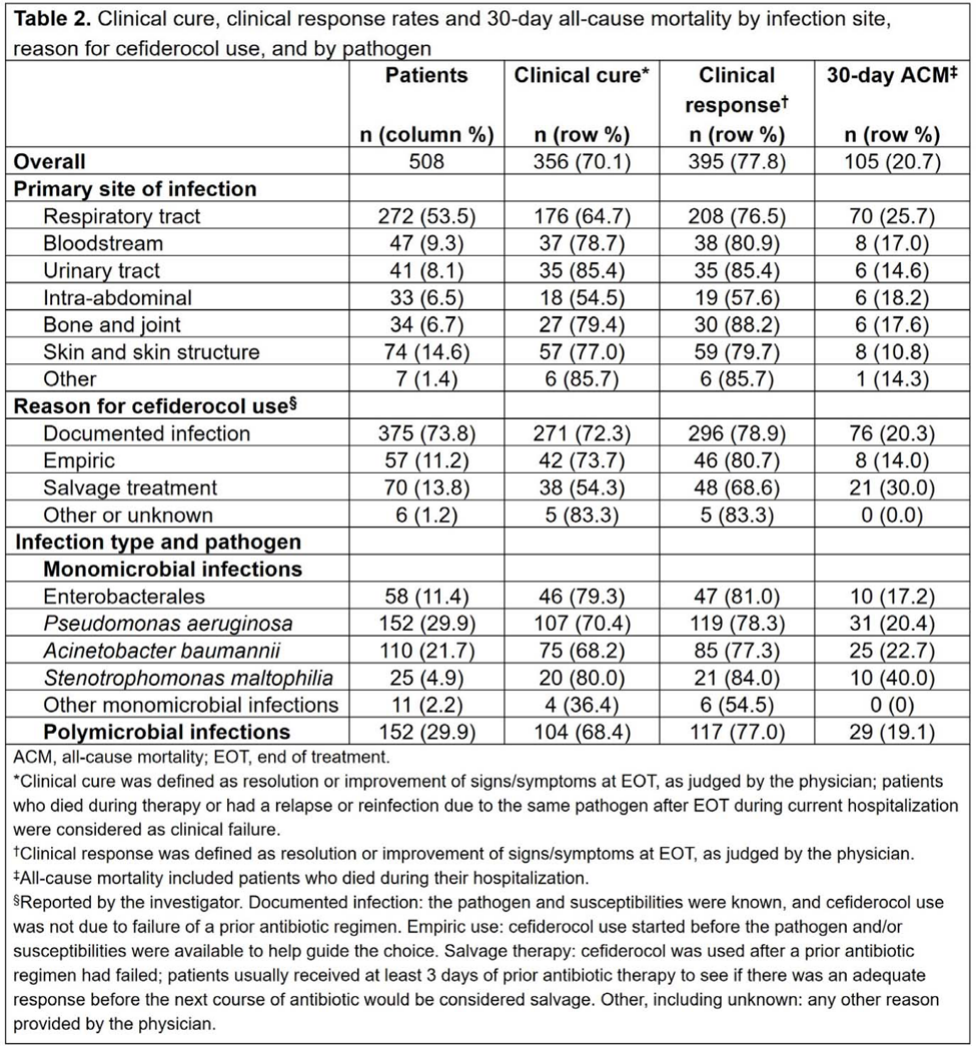

**Methods:**

PROVE was an international, retrospective, observational medical chart review study (November 2020–July 2024). Data were analyzed from hospitalized US patients with confirmed Gram-negative bacterial infections, who received cefiderocol for the first time for ≥72 hours. Baseline demographics, clinical characteristics, cefiderocol use, clinical response, clinical cure, and all-cause mortality (ACM) were assessed.

**Results:**

Median (interquartile range [IQR]) age of patients was 58 (44–67) years and 61.2% were men (Table 1). The three most frequent concomitant conditions were diabetes mellitus (36.0%), chronic pulmonary disease (25.0%), and sepsis/septic shock (23.2%). Cefiderocol was given for a median (IQR) of 10 (7–16) days and as combination therapy in 33.1% of patients. At cefiderocol initiation, 57.3% of patients were in the intensive care unit and 47.6% were receiving organ support. Most patients (93.3%) had ≥1 risk factor for acquiring carbapenem-resistant (CR) Gram-negative bacteria. Respiratory tract infection (RTI) was the most common infection (n=272; 53.5%), followed by skin and skin structure infection (n=74; 14.6%) (Table 2). Overall rates of clinical cure, clinical response, and 30-day ACM were 70.1%, 77.8%, and 20.7%, respectively. Among patients with RTIs, clinical cure and 30-day ACM rates were 64.7% and 25.7%, respectively. Clinical cure rates were 73.7% (95% CI: 62.3–85.1%) when cefiderocol was used empirically and 72.3% (95% CI: 67.7–76.8%) when used for documented infections, whereas clinical cure rate was 54.3% (95% CI: 42.6–66.0%) when used as salvage therapy. Clinical cure rates for patients with Enterobacterales and *Pseudomonas aeruginosa* infections were 79.3% and 70.4%, with respective 30-day ACM rates of 17.2% and 20.4%.

**Conclusion:**

US patients at risk for acquiring CR infections receiving cefiderocol as salvage therapy had a lower clinical cure rate than those treated empirically or for documented infections; thus, earlier cefiderocol treatment prior to clinical decline may be beneficial for such patients.

**Disclosures:**

Cornelius J. Clancy, MD, Merck: Grant/Research Support|Shionogi: Advisor/Consultant Anne Lachiewicz, MD, MPH, Basilea: Grant/Research Support|Pfizer: Grant/Research Support|Shionogi: Grant/Research Support Stefano Verardi, MD, Shionogi BV: Employee Karan Gill, Master of Science, Shionogi BV: Employee Anne Santerre Henriksen, PHD, Shionogi BV: Advisor/Consultant Sean T. Nguyen, PharmD, Shionogi Inc: Employee

